# Belladonna

**Published:** 1914-10

**Authors:** E. S. Goodhue


For Texas Medical Journal.

### Belladonna.

BY E. S. GOODHUE.

I love thee, Belladonna, dear,
Though poor in health am I;
I hold thee in my heart, my own,
As well as in my eye.

With morphine, strychnine, opium,
I ne'er have aught to do;
But, Belladonna, dear, I'll live
To thee forever true!

My puffed conceit thou dost reduce,
My love and dearest fate;
I am thy pupil and I shall
Upon thy power dilate!

Thy principle I know full well,
Is strong and good and pure;
Now for my aching breast thou canst
Full ease and rest secure!

At touch of thee my breathings haste,
My pale cheeks flush with glee;
So, Belladonna, dear, I give
My morbid self to thee!

